# Positron Emission Tomography as a Method for Measuring Drug Delivery to Tumors *in vivo*: The Example of [^11^C]docetaxel

**DOI:** 10.3389/fonc.2013.00208

**Published:** 2013-08-13

**Authors:** Astrid A. M. van der Veldt, Egbert F. Smit, Adriaan A. Lammertsma

**Affiliations:** ^1^Department of Internal Medicine, VU University Medical Center, Amsterdam, Netherlands; ^2^Department of Radiology and Nuclear Medicine, VU University Medical Center, Amsterdam, Netherlands; ^3^Department of Pulmonary Diseases, VU University Medical Center, Amsterdam, Netherlands

**Keywords:** positron emission tomography, radiolabeled anticancer drugs, drug delivery, tumors, [^11^C]docetaxel, lung cancer

## Abstract

Systemic anticancer treatments fail in a substantial number of patients. This may be caused by inadequate uptake and penetration of drugs in malignant tumors. Consequently, improvement of drug delivery to solid tumors may enhance its efficacy. Before evaluating strategies to enhance drug uptake in tumors, better understanding of drug delivery to human tumors is needed. Positron emission tomography (PET) is an imaging technique that can be used to monitor drug pharmacokinetics non-invasively in patients, based on radiolabeling these drugs with short-lived positron emitters. In this mini review, principles and potential applications of PET using radiolabeled anticancer drugs will be discussed with respect to personalized treatment planning in oncology. In particular, it will be discussed how these radiolabeled anticancer drugs could help to develop strategies for improved drug delivery to solid tumors. The development and clinical implementation of PET using radiolabeled anticancer drugs will be illustrated by validation studies of carbon-11 labeled docetaxel ([^11^C]docetaxel) in lung cancer patients.

## Introduction

To date, an increasing number of anticancer drugs is available for treating cancer patients. Nevertheless, resistance to anticancer drugs remains a problem in a substantial number of patients and, consequently, these patients may suffer from drug-induced toxicities without any benefit. Tumor response to anticancer drugs is, amongst others, thought to be directly related to drug concentrations in tumor tissue. Strategies that improve drug delivery to tumors may therefore enhance efficacy of anticancer drugs. Prior to the evaluation of these strategies, better understanding of drug delivery to human tumors is needed. Direct assessment of tumor drug concentrations in cancer patients, however, is challenging, as it requires accessibility to tumors that are usually deeply seated within the body. Positron emission tomography (PET) is an imaging technique that can be used to monitor drug pharmacokinetics non-invasively in patients by radiolabeling drugs of interest with short-lived positron emitters. In this mini review, principles and potential applications of PET using radiolabeled anticancer drugs will be discussed for personalized treatment planning in oncology. Furthermore, development and clinical implementation of radiolabeled anticancer drugs will be illustrated by validation studies of carbon-11 labeled docetaxel ([^11^C]docetaxel) in lung cancer patients. Finally, it will be discussed how these radiolabeled anticancer drugs could help to develop strategies for improved drug delivery to tumors.

## Positron Emission Tomography

### Principles of PET

**Figure 1:**
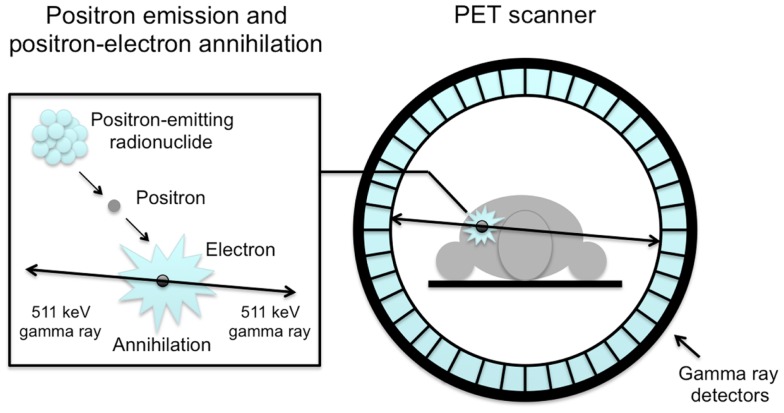
**Schematic illustration of an annihilation reaction and the subsequent coincidence detection**. Positrons released from the nucleus of the radionuclide annihilate with electrons in tissue, releasing two coincidence photons of 511 keV, which are detected by scintillation crystals (blue rectangles). Coincidence detection of annihilated photons identifies a line-of-response and makes it possible to localize the source of the annihilation.

Positron emission tomography is a highly sensitive nuclear imaging technique that enables non-invasive *in vivo* monitoring of dynamic processes (1). PET tracers are molecules of interest that are labeled with a positron emitting radionuclide. Such a radionuclide decays by emission of a positron from its nucleus, which almost immediately results in the simultaneous emission of two gamma rays in opposite direction. For PET imaging, in general short-lived radionuclides, such as carbon-11 [^11^C], fluorine-18 [^18^F], and oxygen-15 [^15^O] are used. A PET scanner usually consists of a ring of detectors and is capable of detecting high-energy gamma rays that are emitted from tissue after intravenous administration of a PET tracer (Figure 1). After reconstruction, data obtained provide information on the 3-dimensional tracer concentration within the body. To date, a PET scanner is combined with an integrated computed tomography (CT) scanner (2), which is used for attenuation correction as well as anatomical localization of tracer uptake.

### Kinetic modeling of PET data

**Figure 2:**
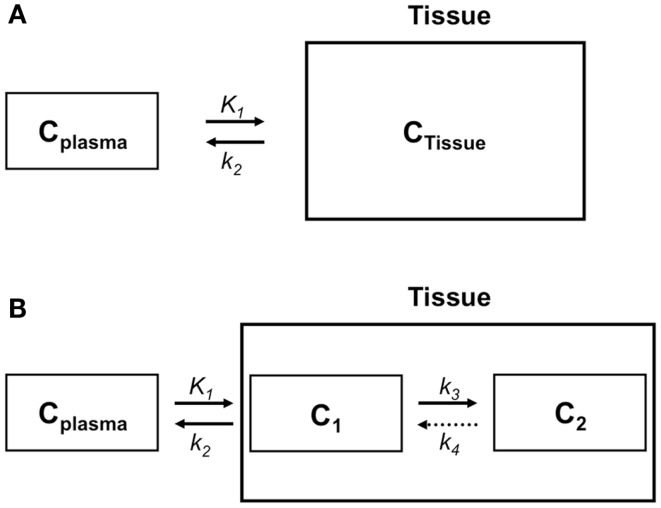
**Compartment models to describe the behavior of a tracer in tissue**. **(A)** Schematic diagram of a single tissue compartment model in which only one tissue compartment can be distinguished, such as in the case of a flow tracer. According to this model, the tracer concentration in tissue (*C*_Tissue_) depends on plasma concentration (*C*_Plasma_), influx from plasma (*K*_1_ or rate constant for transfer from plasma to tissue), and clearance from tissue to plasma (*k*_2_ or rate constant for transfer from tissue to plasma). **(B)** Schematic diagram of a two tissue compartment model. *C*_Tissue_ consists of tracer concentrations in compartments 1 and 2, representing free (*C*_1_) and bound or metabolized tracer (*C*_2_), respectively. Tracer kinetics in tissue are regulated by *C*_Plasma_ and four kinetic rate constants *K*_1_, *k*_2_, *k*_3_, and *k*_4_. *K*_1_ is the rate constant for transport from plasma to tissue, *k*_2_ for transport from tissue to plasma, and *k*_3_ and *k*_4_ are kinetic rate constants describing exchange between the two tissue compartments. For an irreversible two tissue compartment model*k*_4_ = 0.

**Figure 3:**
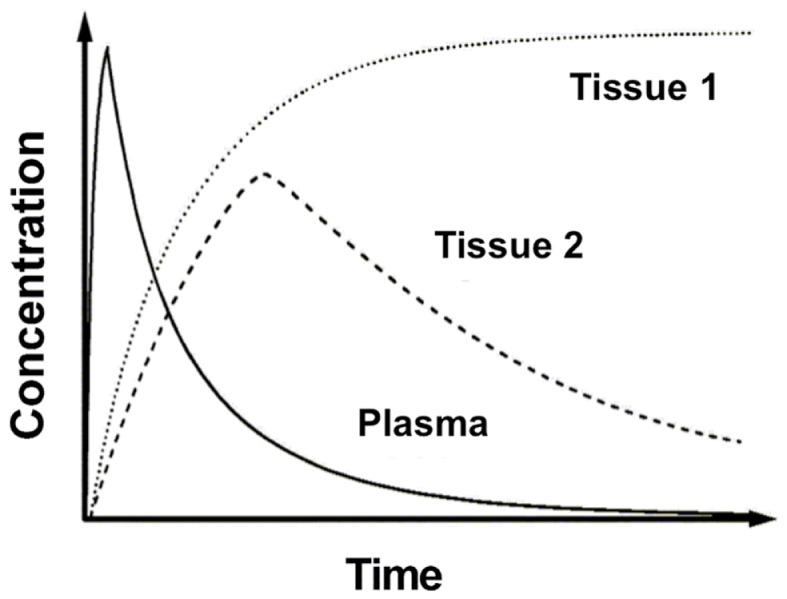
**Time-activity curves**. Example of time-activity curves of radioactivity concentrations in plasma and two different tissues of interest.

**Figure 4:**
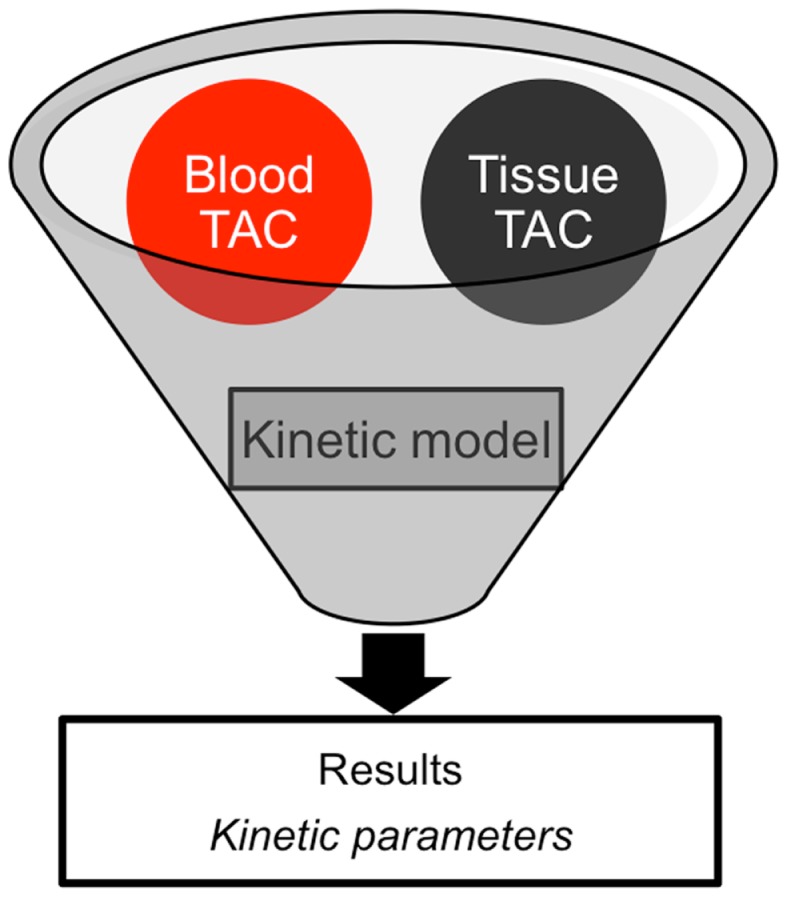
**Schematic diagram of data required for analysis of PET data (TAC: time-activity curve)**.

In clinical practice, a PET image can be extremely useful for diagnosis and staging of cancer. However, absolute quantification of tracer kinetics in tissue is necessary for complete characterization of functional processes *in vivo*. For quantification of tracers, their uptake in tissue needs to be measured as function of time. Therefore, PET data need to be acquired as dynamic rather than static scans. For diagnostic purposes, usually whole body scans are performed, which consist of a series of static scans by moving the scanner bed over multiple bed positions. During a dynamic PET scan, patients are scanned at one bed position and detailed (kinetic) information on a selected part of the body is obtained. As a result, a dynamic scan is limited by the field of view of the PET scanner, which is ∼15–20 cm. Consequently, the tissue of interest needs to be adequately defined prior to acquisition of the PET data. Net tracer uptake in tissue is determined by its delivery, extraction from blood and washout from tissue as function of time. Each tracer has its own distinct behavior *in vivo*, which can be described by tracer kinetic models (3). Several compartmental models have been developed to describe PET data. In Figure 2, schematic diagrams of standard single tissue and two tissue compartment models are presented. The kinetic rate constants in these models can be estimated from dynamic PET data. To this end, a tissue time-activity curve (TAC; Figure 3) is fitted to the appropriate model equation using the arterial plasma TAC as input function, and the best fit then provides estimates of these kinetic parameters (Figure 4). The arterial input function can be obtained from arterial blood sampling using an on-line detection system (4). Arterial blood sampling, however, is an invasive and cumbersome procedure. In principle, the time course of the tracer in a large arterial blood structure, e.g., the aorta, can also be used to generate a non-invasive image derived input function.

### PET imaging in oncology

Over the past decade, clinical applications of PET have expanded, particularly in oncology. To date, 2-deoxy-2-[^18^F]fluoro-d-glucose ([^18^F]FDG) is the most widely used PET tracer for evaluation of cancer. High [^18^F]FDG uptake in tumors is based on altered glucose metabolism in most cancer cells (5). As [^18^F]FDG uptake in tissue is not specific for malignancy and does not provide information on other biological characteristics of tumors, other PET tracers have been developed. For example, 3-deoxy-3-[^18^F]fluorothymidine ([^18^F]FLT) has been developed to measure tumor proliferation (6). In addition, radioactive water ([^15^O]H_2_O) can be used to measure tumor perfusion (7), whereas hypoxia tracers such as [^18^F]fluoroazomycinarabinofuranoside ([^18^F]FAZA) and [^18^F]fluoromisonidazole ([^18^F]FMISO) can be used to determine hypoxic areas in tumors (8). Although these PET tracers may provide additional information on various biological processes in tumors and could be useful for response evaluation, they are not specific enough to predict tumor response to specific anticancer drugs. As an alternative, anticancer drugs can be labeled with positron emitters. Using PET, these radiolabeled drugs can then be used to monitor drug pharmacokinetics in patients non-invasively. As tumor response to anticancer drugs is thought to be directly related to drug concentrations in tumor tissue, uptake of radiolabeled anticancer drugs in tumors may predict treatment outcome. Preliminary PET studies using F-18 labeled 5-fluorouracil ([^18^F]5-FU; (9, 10)), tamoxifen ([^18^F]fluorotamoxifen; (11)), and C-11 labeled docetaxel ([^11^C]docetaxel; (12)) showed that high tumor uptake of the radiolabeled anticancer drug was associated with improved tumor response following corresponding therapy. These studies suggest that radiolabeled anticancer drugs may be useful for prediction of outcome prior to start of treatment. Consequently, an increasing number of anticancer drugs has now been radiolabeled including radiolabeled cytotoxic agents (e.g., [^11^C]temozolomide, [^18^F]5-fluorouracil, and [^11^C]docetaxel), selective hormone receptor modulators (e.g., [^18^F]fluorotamoxifen), tyrosine kinase inhibitors (TKIs, e.g., N-[^11^C]methylimatinib, [^11^C]sorafenib, and [^11^C]erlotinib), and monoclonal antibodies [Mabs, e.g., [^89^Zr]cetuximab, [^89^Zr]trastuzumab, and [^89^Zr]bevacizumab; (10, 11, 13–20)].

## Development of Radiolabeled Anticancer Drugs

For the development of radiolabeled anticancer drugs, a complex, and expensive research infrastructure is required: a cyclotron for production of positron emitters, an on-site good manufacturing practice laboratory for synthesis of the tracer, a PET/CT scanner for acquisition of images, an on-line blood sampler in case of arterial blood sampling, an on-site laboratory for measurements of radioactivity concentrations and radioactive metabolites in plasma, and dedicated computers and software to analyze and quantify acquired PET data. In addition, these facilities need to be staffed by qualified personnel including a cyclotron operator, a chemist who synthesizes the PET tracer, a radiopharmacist who is responsible for quality control of the tracer production, a technologist for acquiring PET images, a (nuclear medicine) physician who is clinically responsible for the patient as well as for arterial blood sampling, a chemist for analyzing blood samples during PET scanning, and a physicist who is responsible for acquisition protocols and data analyses. The short half-lives of most PET tracers require that these facilities and personnel are located and working in the same building at very close proximity. Besides these logistic issues, the use of PET and radiolabeled anticancer drugs can be limited by technical issues including complex tracer synthesis and the spatial resolution of the scanner. Before implementation of a new PET tracer in the clinic, technical, and biological validation of the tracer is required. To this end, the optimal patient population should be selected based on patient characteristics and technical issues.

## The Example of [^11^C]Docetaxel Pet in Lung Cancer Patients

### Docetaxel

The cytotoxic agent docetaxel is a taxane, a class of drugs consisting of microtubule stabilizing agents that function primarily by interfering with microtubular dynamics, inducing cell cycle arrest and apoptosis (21). In clinical practice, docetaxel is administered as a 1-h intravenous infusion, usually given at a dose of 75 or 100 mg m^−2^ in a three-weekly regimen. In 1996, docetaxel was first approved for the treatment of anthracycline-refractory metastatic breast cancer. Thereafter, the drug was registered as monotherapy as well as in combination strategies for the treatment of several advanced malignancies including hormone refractory metastatic prostate cancer, gastric adenocarcinoma, head and neck cancer, and non-small cell lung cancer (NSCLC) (21). In these malignancies, docetaxel has shown clinical efficacy, including tumor response and improved survival. Nevertheless, failure of docetaxel therapy occurs and patients are often subjected to docetaxel related toxicities without gaining benefit.

### Labeling of docetaxel

**Figure 5:**
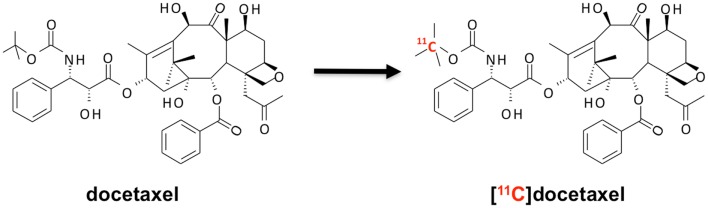
**Synthesis of [^11^C]docetaxel**. [^11^C]docetaxel is synthesized by replacing a stable carbon atom by carbon-11 (22, 23), so that chemical properties of stable and labeled compound are exactly the same.

Docetaxel has been radiolabeled with the radionuclide carbon-11 (22, 23). As a stable carbon atom is replaced by carbon-11 (Figure 5), the chemical structure of the tracer [^11^C]docetaxel is identical to that of the drug docetaxel. Hence, pharmacokinetics of tracer and drug are identical. As the specific activity of [^11^C]docetaxel is approximately 10 GBq μmol^−1^, which contains 30 μg docetaxel for a typical administration of 370 MBq [^11^C]docetaxel, only 0.02% of a therapeutic dose of docetaxel is administered for PET. As a result, [^11^C]docetaxel microdosing prevents patients from drug-induced toxicities that are associated with therapeutic doses. The following paragraphs describe successive steps in the validation of [^11^C]docetaxel for use in lung cancer patients.

### Biodistribution of [^11^C]docetaxel in rats

**Figure 6:**
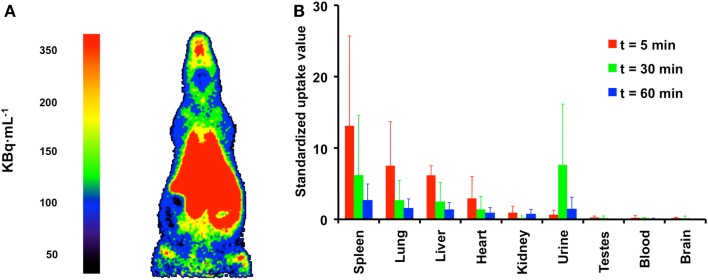
**Biodistribution of [^11^C]docetaxel in healthy male rats**. **(A)** PET image showing the biodistribution of [^11^C]docetaxel in a male rat. This image was obtained using a high resolution research tomograph (HRRT) with a spatial resolution of about 2.5 mm. Red indicates the highest [^11^C]docetaxel uptake. **(B)** Standardized uptake values of [^11^C]docetaxel in organs as obtained from dissection studies. Standardized uptake values were calculated as tissue radioactivity concentration normalized for injected dose and body weight.

In preparation of humans studies, the biodistribution of [^11^C]docetaxel in healthy rats was investigated (24). This preclinical study was needed to obtain an initial estimate of the expected radiation dose in humans, which in turn was required for obtaining ethics permission to conduct human studies. The biodistribution of [^11^C]docetaxel was determined in healthy male rats at 5, 15, 30, and 60 min after injection. This preclinical study showed the highest [^11^C]docetaxel uptake in spleen, followed by urine, lung and liver, whereas brain and testes showed the lowest uptake (Figure 6). Within less than 5 min, [^11^C]docetaxel essentially had cleared from blood and plasma. As the estimated effective dose in humans extrapolated from this rat study was 5.4 μSv MBq^−1^, the use of [^11^C]docetaxel in humans was considered to be safe.

### Biodistribution of [^11^C]docetaxel in humans

**Figure 7:**
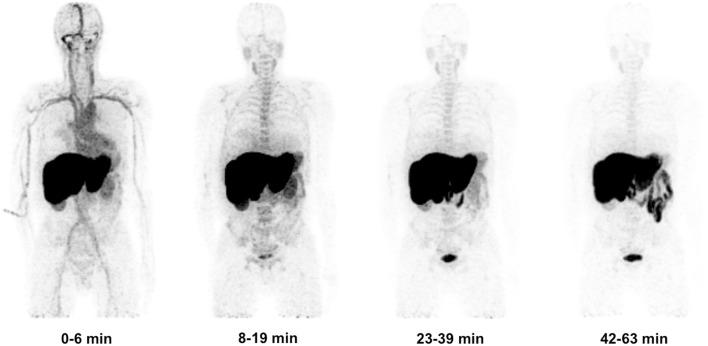
**Biodistribution of [^11^C]docetaxel in patients**. Four successive [^11^C]docetaxel whole body PET scans showing that [^11^C]docetaxel first accumulates in liver, before being excreted into bile and ultimately into intestine. Because of high [^11^C]docetaxel uptake in the liver, these projections do not show the high uptake of [^11^C]docetaxel in the gall bladder (25).

Following the preclinical study in rats, both biodistribution and actual human radiation dosimetry of [^11^C]docetaxel was determined in seven patients with solid tumors using whole body PET/CT scans (25). Gall bladder and liver showed high [^11^C]docetaxel uptake, whilst uptake in brain and normal lung was low (Figure 7). In the liver, the percentage injected dose at 1 h was 47 ± 9%. In addition, [^11^C]docetaxel was rapidly cleared from plasma and no radiolabeled metabolites were detected. The effective dose of [^11^C]docetaxel was 4.7 μSv MBq^−^1, which was comparable to the estimated effective dose in rats. In contrast to the preclinical study in rats, [^11^C]docetaxel showed low uptake in human lungs. As a result, [^11^C]docetaxel could be a useful tracer for tumors in the thoracic region.

### Quantification of tumor uptake

**Figure 8:**
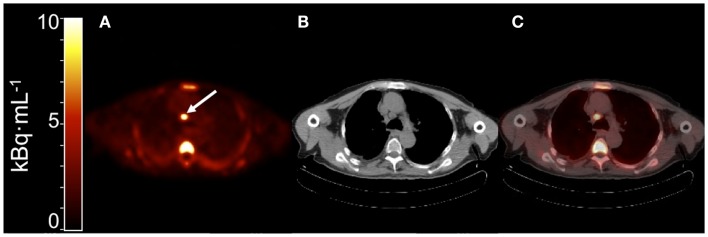
**(A)** Summed PET image of [^11^C]docetaxel uptake from 10 to 60 min post injection showing a mediastinal metastasis with increased uptake [arrow; (12)]. **(B)** Corresponding CT image. **(C)** PET-CT fusion image.

Although uptake of [^11^C]docetaxel in normal tissues may be interesting, its uptake in tumor tissue is more important. The feasibility of quantitative [^11^C]docetaxel PET scans was evaluated in patients with lung cancer (Figure 8). In addition, it was investigated whether [^11^C]docetaxel kinetics were associated with tumor perfusion or tumor size. In this study, 34 lung cancer patients underwent dynamic PET/CT scans using [^11^C]docetaxel and [^15^O]H_2_O (12). For quantification of [^11^C]docetaxel kinetics, the optimal tracer kinetic model was determined. Tumor kinetics of [^11^C]docetaxel were irreversible and could be quantified using Patlak graphical analysis. Furthermore, it was shown that reproducible quantification of [^11^C]docetaxel kinetics in tumors was possible using a non-invasive image derived input function. In tumors, the net rate of influx (*K*_i_) of [^11^C]docetaxel was variable and strongly related to tumor perfusion, but not to tumor size. Finally, effects of dexamethasone administration on drug uptake in tumors were investigated, as corticosteroids are potent inducers of the drug efflux transporter ABCB1. Prior to administration of therapeutic doses of docetaxel, all patients are premedicated with corticosteroids, as this reduces incidence and severity of docetaxel induced fluid retention and hypersensitivity reactions significantly (26, 27). In this dynamic PET study, the first 24 patients were premedicated with dexamethasone, whereas the last 10 patients were not. In dexamethasone premedicated patients, uptake of [^11^C]docetaxel in tumors was significantly lower than in patients without premedication, indicating that co-medication may affect accumulation of drugs in tumor tissue. Finally, in a subgroup of patients who subsequently received docetaxel therapy, high tumor uptake of [^11^C]docetaxel was related with improved tumor response (12, 28), suggesting that the observed variation in [^11^C]docetaxel kinetics between tumors may reflect differential sensitivity to docetaxel therapy.

### Validation of the microdosing concept

[^11^C]docetaxel microdosing protects patients from toxicities that are associated with therapeutic doses of docetaxel. However, pharmacokinetics of [^11^C]docetaxel at tracer doses may be different from those at therapeutic doses, as the latter can significantly affect uptake of radiolabeled anticancer drugs in normal organs as well as in tumors (29–32). Therefore, the microdosing concept was validated for [^11^C]docetaxel in another study (28). The research question to be addressed was whether a PET study using a tracer dose of [^11^C]docetaxel could predict tumor uptake of unlabeled docetaxel during a therapeutic infusion. For this purpose, docetaxel naïve lung cancer patients underwent two [^11^C]docetaxel PET scans, one after a bolus injection of a tracer dose [^11^C]docetaxel and another during a combined infusion of a tracer dose [^11^C]docetaxel and a therapeutic dose of docetaxel (75 mg m^−2^). Compartmental and spectral analyses were used to quantify [^11^C]docetaxel tumor kinetics. In addition, [^11^C]docetaxel PET measurements were used to estimate the area under the curve of therapeutic doses of docetaxel in tumors. At 90 min, the accumulated amount of docetaxel in tumors was<1% of the total infused dose of docetaxel, indicating that only a small amount accumulates in tumors. In addition, the uptake of therapeutic doses in tumors was related to the uptake of [^11^C]docetaxel during the microdosing scan, indicating that [^11^C]docetaxel PET can be used to predict tumor uptake of docetaxel during chemotherapy.

### Combination therapy

Within the context of combination therapy, effects of the anti-angiogenic drug bevacizumab on tumor perfusion and [^11^C]docetaxel uptake in lung tumors were investigated in NSCLC patients (33). Bevacizumab is a humanized monoclonal antibody that targets circulating vascular endothelial growth factor (VEGF) and subsequently prevents binding of VEGF to its receptors. Combined with chemotherapy, bevacizumab has been approved for the treatment of several advanced malignancies including NSCLC (34). It is assumed that anti-angiogenic drugs, such as bevacizumab, transiently normalize abnormal tumor vasculature and contribute to improved delivery of subsequent chemotherapy (35). To investigate this concept, a study was performed in NSCLC patients using PET and [^11^C]docetaxel. Within 5 h, a therapeutic dose of bevacizumab reduced both perfusion and [^11^C]docetaxel uptake in NSCLC. These effects persisted after 4 days and were not associated with significant changes in heterogeneity of [^11^C]docetaxel uptake in tumors. Reduction in [^11^C]docetaxel delivery to tumors was accompanied by rapid reduction in circulating levels of VEGF. The clinical relevance of these findings is notable (36–38), as there was no evidence for substantial improvement in drug delivery to tumors after administration of bevacizumab. This study highlights the ability of PET to potentially optimize scheduling of (anti-angiogenic) drugs.

## Conclusion

PET using radiolabeled anticancer drugs may help to reveal the underlying mechanisms of treatment failure in cancer patients. In particular, this technology enables assessment of accumulation of drugs in human tumors and, in turn, prediction of treatment outcome. However, development of radiolabeled drugs faces several caveats on the path from development to clinical implementation, as it can be very challenging due to technical, logistical, financial, and/or patient related issues. To facilitate clinical implementation of radiolabeled drugs, a step-wise approach needs to be applied. In this regard, the step-wise validation of [^11^C]docetaxel in lung cancer patients provides a framework for investigating the PET microdosing concept for other radiolabeled anticancer drugs. The [^11^C]docetaxel PET studies have shown that only a small amount of docetaxel accumulates in tumor tissue, which is further decreased by co-medication (dexamethasone) and other anticancer drugs (bevacizumab). In addition, it is conceivable that drug delivery to tumors is also dependent on the localization of tumors in de body, as drug delivery may differ between organs (e.g., brain versus liver). In this way, PET using radiolabeled anticancer drugs may provide insight into drug delivery to human tumors and may facilitate rational treatment choices that are tailored to improve drug delivery to tumors.

## Conflict of Interest Statement

The authors declare that the research was conducted in the absence of any commercial or financial relationships that could be construed as a potential conflict of interest.
